# Cortisol levels in rural Latina breast cancer survivors participating in a peer-delivered cognitive-behavioral stress management intervention: The *Nuevo Amanecer-II RCT*

**DOI:** 10.1016/j.cpnec.2022.100153

**Published:** 2022-06-24

**Authors:** Cathy Samayoa, Veronica Santana-Ufret, Jasmine Santoyo-Olsson, Paula D. Strassle, Anita Stewart, Jackie Bonilla, Cristian Escalera, Rebecca Margarita Mendez, Leticia Márquez-Magaña, Carmen Ortiz, Rachel M. Ceballos, Anna Maria Nápoles

**Affiliations:** a Department of Epidemiology and Biostatistics, University of California San Francisco, 550 16th St., San Francisco, CA, 94143, USA; b Health & Equity Research Lab, Department of Biology, San Francisco State University, 1600 Holloway Ave, San Francisco, CA, 94132, USA; c Division of Intramural Research, National Institute on Minority Health and Health Disparities, National Institutes of Health, 9000 Rockville Pike, Building 3, Floor 5, Room E08, Bethesda, MD, 20892, USA; d Division of General Internal Medicine, Department of Medicine, University of California San Francisco (UCSF), 3333 California St., Suite 335, San Francisco, CA, 94143, USA; e University of California San Francisco, Institute for Health & Aging, Center for Aging in Diverse Communities, 490 Illinois Street, 12th Floor, Box 0646, San Francisco, CA, USA, 94158; f Círculo de Vida Cancer Support and Resource Center, 2601 Mission St, Suite 702, San Francisco, CA, 94110, USA; g Fred Hutchinson Cancer Research Center, 1100 Fairview Ave, Seattle, WA, 98109, USA

**Keywords:** Cortisol, Cortisol awakening response, Stress, Biomarkers, Breast cancer, Hispanic/latina, Disparities, Hypothalamic-pituitary-adrenal axis

## Abstract

**Background:**

Compared to their White counterparts, Latina breast cancer survivors have poorer survival rates and health-related quality of life, and higher rates of depression and anxiety which may be a result of chronic stress. Chronic stress impacts the hypothalamic-pituitary-adrenal (HPA) axis, resulting in cortisol dysregulation which may be associated with breast cancer survival. However, cortisol levels and cortisol profiles of Latina breast cancer survivors are poorly characterized due to their underrepresentation in biomedical research.

**Objective:**

The objective of this study was to describe cortisol levels and patterns of cortisol secretions in rural Latina breast cancer survivors participating in an RCT study of *Nuevo Amanecer-II*, an evidence-based peer-delivered cognitive behavioral stress management intervention.

**Methods:**

Participant-centered recruitment and collection strategies were used to obtain biospecimens for cortisol analysis. Nine saliva samples (3/day for 3 days) and a hair sample were obtained at baseline and 6-months (3-months post-intervention). We describe cortisol levels and profiles, explore correlations of biomarkers with self-report measures of stress and psychological distress, and compare women who received the intervention with a delayed intervention group on biomarkers of stress. Mean hair cortisol concentration (HCC) was used to assess chronic stress. Based on daily measures of cortisol (awakening, 30 min post-awakening, and bedtime), we calculated three summary measures of the dynamic nature of the cortisol awakening response (CAR): 1) the CAR slope, 2) whether CAR demonstrates a percent change ≥40, and 3) total daily cortisol output (AUCg). Linear and log-binomial regression, accounting for multiple samples per participant, were used to compare cortisol measures at 6-month follow-up by treatment arm.

**Results:**

Participants (n = 103) were from two rural California communities; 76 provided at least one saliva sample at baseline and follow-up and were included in the analysis. At baseline, mean age was 57 years, mean years since diagnosis was 2 years, 76% had a high school education or less, and 34% reported financial hardship. The overall median CAR slope was 0.10, and median cortisol AUCg (in thousands) was 11.34 (range = 0.93, 36.66). Mean hair cortisol concentration was 1751.6 pg/mg (SD = 1148.6). Forty-two percent of samples had a ≥40% change in CAR. We found no statistically significant correlations between the cortisol measures and self-reported measures of stress and psychological distress. At follow-up, no differences were seen in HCC (mean difference between intervention and control: −0.11, 95% CI -0.48, 0.25), CAR slope (0.001, 95% CI -0.005, 0.008), cortisol AUCg (−0.15, 95% CI -0.42, 0.13), or ≥40% change in CAR (prevalence ratio 0.87, 95% CI 0.42, 1.77) between treatment arms.

**Conclusion:**

Our findings of flattened cortisol profiles among more than half of the sample suggest potential HPA-axis dysregulation among rural Spanish-speaking Latina breast cancer survivors that merits further study due to its implications for long-term survival.

**Trial registration:**

http://www.ClinicalTrials.gov identifier NCT02931552.

## Highlights

• Latina breast cancer survivors displayed flattened cortisol profiles.
• Hair cortisol levels, a measure of chronic stress, were found to be elevated.
• Stress biomarkers and self-report measures did not substantially correlate.
• Latina breast cancer survivors are vulnerable to the repercussion of HPA-axis dysregulation.

## 1 Introduction

Breast cancer survivors report elevated levels of stress throughout survivorship [1]. Latina breast cancer survivors are vulnerable to stress due to language barriers, inadequate health insurance, financial challenges, perceived discrimination, and obstacles navigating the health care system [2]. Spanish-speaking Latina breast cancer survivors in particular are at high risk of stress due to limited access to adequate cancer care and other structural barriers associated with health disparities among rural populations [3,4].

Some evidence exists for a link between chronic stress and cancer incidence and progression [[5], [6], [7]], although these associations and their strength vary by cancer type. In the case of breast cancer, several biological mechanisms have been postulated. Converging in vitro and animal studies support that cortisol is associated with breast cancer progression through glucocorticoid signaling [[8], [9], [10]] suppresses cell-mediated immune responses [11], increases tumor volume, and promotes therapy resistance and metastasis, in part, due to activation of the glucocorticoid receptor which can decrease the efficacy of chemotherapy [[12], [13], [14]], resulting in a worse prognosis. Elevated cortisol levels and dysregulation of cortisol secretion associated with chronic stress may contribute to breast cancer initiation, progression, therapy resistance, and recurrence [15]. Several landmark studies have shown an association between cortisol patterns and cancer symptomology and survival in breast cancer patients [[16], [17], [18]]. Specifically, studies have found that a flat slope predicted earlier mortality and is associated with poor health outcomes [18,19].

Latina breast cancer survivors have worse survival rates than white women [20], worse health-related quality of life, and higher levels of pain and anxiety associated with their diagnosis [2]. Thus, higher levels of chronic stress and subsequent effects on elevated cortisol levels among Latina breast cancer survivors provide a potential mechanistic explanation for their disparate health outcomes when compared to other populations. However, the diurnal cortisol rhythm profiles of Latina breast cancer survivors have not been characterized.

Cognitive-behavioral stress management (CBSM) interventions have been shown to improve self-reported stress symptomatology, anxiety, and quality of life [21]. Evidence of the effects of such interventions on stress biomarkers have been mixed [5,22]. Furthermore, studies of this nature have historically focused on predominantly white breast cancer survivors.

The primary aim of this study was to characterize the diurnal cortisol rhythm profiles of Latina breast cancer survivors living in rural areas, using salivary and hair cortisol measures. The sample consists of women who participated in a randomized-controlled (RCT) trial investigating the effects of *Nuevo Amanecer-II*, an evidence-based peer-delivered cognitive-based stress management (CBSM) intervention [23]. For the primary aim (aim 1), we hypothesized that due to their intersecting vulnerabilities (e.g., ethnic minority status, limited English proficiency, low-income, and rural residence), women would demonstrate cortisol levels and patterns consistent with chronic stress. In aim 2, we examined correlations of the cortisol measures with self-reported stress and psychological distress, hypothesizing that cortisol and self-report measures would be positively correlated. Finally, in aim 3 which were exploratory analyses (since this aim was not designed as a part of the original RCT in which this ancillary study was embedded), we assessed associations between intervention or delayed intervention control group assignment with cortisol measures of stress (we hypothesized that women in the stress management intervention group would demonstrate better cortisol profiles than women in the delayed intervention control group). Examining cortisol levels and patterns of cortisol secretions among Latina breast cancer survivors in a stress management RCT can provide mechanistic insights to better understand how stress gets under the skin and potentially contributes to cancer health disparities.

## 2 Methods

### 2.1 Data source and patient population

This work leveraged the parent Nuevo Amanecer-II (NA-II) study, a 6-month longitudinal randomized controlled trial (RCT) with two arms, a CBSM intervention group and a delayed intervention control group. This ancillary biomarker study included participants from two of the RCT study sites who consented to providing biomarkers. Participants were Spanish-speaking Latina breast cancer survivors diagnosed with non-metastatic primary breast cancer and residing in rural areas served by the study's community partners. Women with metastatic disease and women with plans to move within 6-months were excluded.

Participants were recruited from 2016 to 2018 by two of the study's community partners: Cancer Resource Center of the Desert (CRCD) in El Centro, CA, and Kaweah Delta Health Care District in Visalia, CA. Both are community-based organizations that serve Latino populations in communities with a large agribusiness. Recruitment of participants to the parent Nuevo Amanecer-II study is described elsewhere [24]. Eligibility criteria for the RCT were broad by intention since this was an effectiveness trial in anticipation of broader dissemination of the stress management intervention being tested: 1) Spanish-speaking Latina (self-identified); 2) diagnosis of Stage 0 to IIC primary breast cancer; and 3) residing in rural California communities in Imperial, Tulare, or Santa Cruz/Monterey. For this ancillary study, referred henceforth as the cortisol study, all women from the Visalia (Tulare County) and El Centro (Imperial County), California, sites were eligible for inclusion (n = 103). Women were excluded from the analysis if they did not complete their 6-month follow-up assessment or failed to provide at least one saliva sample at the 6-month follow-up assessment.

This study was approved by the Institutional Review Board (IRB) at San Francisco State University (protocol #E13-169), the University of California, San Francisco (protocol #16-18737), and the Kaweah Delta Health Care District IRB (protocol # 20160434006). Written informed consent was obtained concurrently for this study and the parent NA-II study. The parent study trial is registered at http://www.ClinicalTrials.gov (NCT02931552).

### 2.2 Intervention

A detailed description of the NA-II peer-delivered CBSM intervention can be found elsewhere [23,24]. Briefly, the intervention was a 10-week CBSM peer-delivered program. Each week, trained compañeras (interventionists) delivered an in-person, hands-on 90-min module to teach and reinforce the concepts and stress management skills of the NA-II intervention. In-person meetings took place at participants’ homes or other locations based on participant preference.

### 2.3 Biospecimen collection

To collect the salivary and hair biospecimens, participant-centered strategies (e.g., demonstration videos, low-literacy written instructions with photos, telephone call reminders, assistance from a community recruiter) were developed to overcome barriers to biospecimen collection; these are described in detail elsewhere [25]. Briefly, community recruiters from the partner community organizations were trained to facilitate the biospecimen collection process. Saliva samples and hair specimens were collected at baseline, and 6-month follow-up from both intervention and control group (prior to initiation of the delayed intervention) women. Collection kits containing materials for the collection of cortisol salivary samples and hair samples were assembled in the Health & Equity Research Lab at San Francisco State University. Community recruiters distributed sample collection kits in person, performed collection of the hair sample, and instructed participants on self-collection of salivary samples.

For salivary cortisol samples, participants were instructed to passively drool into a cryovial (WHEATON CryoELITE vial, DWL Life Sciences, Millville, NJ) and provide 1.5 mL of saliva at 3-time points: at awakening, 30 min post-awakening, and bedtime for three consecutive days, for a total of nine saliva samples. Participants recorded the time of collection for each sample using a time log provided. To avoid blood and debris contamination, participants were instructed to avoid teeth brushing and eating 1 h before salivary sample collection.

After collecting salivary samples, participants were instructed to store the saliva sample in their home refrigerator (4 °C) until the community recruiter returned to pick up the sample. Samples were then kept refrigerated (4 °C) at the community-based organization (CBO) partner study site (for a maximum of approximately 3 weeks). Samples were then transported in a cooler to San Francisco State University by the study investigator, logged, and stored in a freezer (−20 °C) until further processing.

For hair collection, the kit included two small rubber bands, an index card depicting the orientation of the hair for proper identification of root end, an adhesive strip, a glove, and an envelope for storage of hair samples. For hair sample collection, hair strands approximately the thickness of a pencil eraser were collected from the root at baseline and 6-months. Participants were instructed to avoid coloring hair for at least 2 weeks before a sample was taken [26].

### 2.4 Processing of hair and saliva

Samples containing approximately 1.5 mL of saliva were spun down for 15 min at 4500 rpm. The supernatant was transferred to a 1.5 ml Eppendorf tube and stored at −20 °C.

Hair samples were cut from the root end (1.5 cm) and washed in methanol three times for cleaning [27]. Hair was dried overnight, placed in round-bottom tubes with two stainless steel 5 mm balls, and pulverized using a mixer mill (Mixer Mill Retsch MM 400, Hann, Germany) set to 30 Hz for 5 min. Once pulverized, 10 mg of hair was transferred to a glass vial (1.0 DR, VWR). If 10 mg was not present, the maximum amount of pulverized hair was used and noted [28].

### 2.5 Salivary cortisol quantification

Cortisol quantification was performed in triplicate with 50 μL of saliva using a commercially available cortisol ELISA (catalog # 11-CORHU-E01-SLV, ALPCO, Salem, NH) as previously published by the Health & Equity Research Lab at SFSU [29]. Quantification analysis was carried out per the manufacturer's instructions. Cortisol concentration was calculated in ng/ml based on a 450 nm absorption five-parameter linear regression analysis using a multi-mode microplate plate reader (Synergy HTX multi-mode reader, BioTek, Winooski, Vermont) and accompanying microplate data analysis software (Gen 5 version 2.04, BioTek). Triplicate samples with an intra-assay coefficient of variability (CV) of less than 20% were included in the analysis. The limit of the detection range was 1 ng/mL-100 ng/mL. Salivary cortisol concentrations were assessed for awakening, 30 min post-awakening, and bedtime levels. Based on these measures, a variety of characteristics of the daily diurnal cortisol secretion and daily total cortisol output were calculated based on previous work (described below) [[30], [31], [32], [33]]. Salivary cortisol measures are reported in ng/mL.

### 2.6 Hair cortisol quantification

In this study, 1.5 cm of hair from the root was used to assess physiological stress in the past 6 weeks [27,34]. Hair cortisol concentrations were calculated from a one-time sample collection.

To extract cortisol from the hair, 1 mL of methanol was added to the glass vial containing pulverized hair. These samples were then incubated at 56 °C in a water shaker (C76 water bath shaker, New Brunswick Scientific) and for an average of 17 h. The supernatant, which contains cortisol in methanol was transferred into a 1.5 ml Eppendorf tube and centrifuged for 30 s to further separate any remaining hair particles. Approximately 700 mL of supernatant was again transferred into a new 1.5 mL Eppendorf tube, and the exact volume was recorded for use in the calculation step. Samples were subjected to evaporation at 38 °C under centrifuge vacuum conditions (CentriVap concentrator, Labconco, Kansas City, MO), for 3 h. The remaining solid/precipitate was resuspended in 250 μL of Standard A, a solution containing 0 ng/mL of cortisol, or negative control, from the commercially available ELISA kits (catalog # 11-CORHU-E01-SLV, ALPCO, Salem, NH). Hair cortisol concentrations (HCC) are reported in pg/mg.

### 2.7 Measures

#### 2.7.1 Cortisol measures

Dynamic cortisol secretion patterns can be detected using both hair and salivary samples. Salivary cortisol has been used widely to quantify stress and HPA axis function through characterization of diurnal cortisol rhythm (DCR) and cortisol awakening response (CAR) patterns [35]. Using awakening, 30-min post-awakening, and bedtime salivary samples, investigators can explore different aspects of the cortisol secretion pattern. Circulating cortisol is integrated into the hair over time, thus elevated levels of hair cortisol are indicative of chronic stress [34].

Several components of HPA axis function were measured and calculated based on expert consensus guidelines [36]. Salivary cortisol measures based on the three daily measures of cortisol (awakening, 30 min post-awakening, and bedtime) included three key summary measures of the dynamic nature of the cortisol awakening response (CAR): 1) the CAR slope, 2) whether CAR demonstrates a percent change ≥40, and 3) total daily cortisol output (AUCg). One hair cortisol measure was included, for a total of four cortisol measures.

The first salivary cortisol measure, the CAR slope, aims to capture the daily change in cortisol concentration from awakening to peak (30-min post-awakening) cortisol secretion. CAR slope was calculated as the change in cortisol concentration between the first (awakening) and second (30-min post-awakening) sample, divided by the actual difference in time between sample collection (usually 30 min). A positive and larger CAR slope value represents a healthy cortisol diurnal profile normal stress reactivity, while a smaller or negative value indicates dysregulated patterns, often referred to as a blunted or flattened CAR [36].

For the second salivary measure, the daily percent change in CAR was calculated as the difference between the 30-min post-awakening sample and awakening sample (e.g., CAR slope), divided by the cortisol concentration at awakening. This measure has the advantage that it takes into account the awakening value, which is important because an inverse relationship between awakening and peak cortisol levels is typical (i.e., higher awakening samples are often inversely related to lower peak cortisol levels) [36]. Based on prior literature, we derived a dichotomous variable ≥40% change in daily CAR if the percent change from awakening to peak exceeded 40%. A ≥40% change in CAR represents a favorable stress reactivity profile, whereas a <40% change represents an abnormal profile and is characteristic of CAR non-responders [32,33,37].

The third salivary measure, Area Under the Curve with respect to ground (AUCg) represents an important measure of total post-awakening salivary cortisol output, irrespective of the CAR profile [38]. The AUCg measure integrates all three daily measures of salivary cortisol concentrations (at awakening, 30-min post-awakening, and bedtime). A larger daily AUCg represents higher amounts of salivary cortisol secretion post-awakening. A larger AUCg value represents more cortisol output throughout the waking period.

The fourth and final cortisol measure was hair cortisol concentration (HCC), which is an indicator of the chronic exposure to cortisol experienced during approximately the previous 6 weeks. Cortisol circulating in the blood stream is incorporated into the hair over time [27,34] and cortisol concentrations can be quantified using hair samples. In this study, HCC was calculated as picograms of cortisol per milligrams of hair. A higher value indicates more exposure to circulating concentrations of cortisol over the given period.

#### 2.7.2 Self-reported measures of stress and psychological distress

Self-reported measures of stress (perceived stress) and psychological distress (anxiety, depressive symptoms, health distress) were assessed by surveys completed in Spanish at baseline and 6-months (approximately 3 months post-intervention in the treatment arm and prior to receipt of the delayed intervention among control group women). Perceived stress was assessed with the Spanish version of the 10-item Perceived Stress Scale (PSS-10) [39]. Anxiety was measured using a scale from the Brief Symptom Inventory (BSI) [40]. Depressive symptoms were measured using the Spanish version of the PHQ-8 [41]. Health distress was measured using the Spanish version of the 4-item Health Distress scale developed by the Stanford Patient Education Research Center [42]. For all self-report measures, a higher score indicates more stress or psychological distress. In-depth descriptions of these measures and their psychometric characteristics are reported elsewhere [23].

#### 2.7.3 Demographic and breast cancer characteristics

Demographic measures were obtained at the NA-II parent study baseline assessment and included self-reported age, language read and spoken, education, ethnicity, national origin, years residing in the United States, employment, and experience with financial hardship in the past year. Breast cancer diagnostic and treatment variables were obtained using medical records and included breast cancer type, years since last cancer diagnosis, stage at diagnosis, and type of treatment received [24].

### 2.8 Data analysis

Women who were included in the Cortisol Study analyses and those who were dropped from analyses because they did not complete their 6-month follow-up survey or did not provide at least one saliva sample at the 6-month follow-up were compared on demographic and breast cancer characteristics using Fisher's exact (categorical) and Wilcoxon-Mann-Whitney (continuous) tests. Fisher's exact test examined differences by treatment arm in participant characteristics. Descriptive statistics (means, medians, and proportions) were used to characterize the participants’ baseline characteristics.

Correlation between baseline cortisol biomarker measures and self-report measures of stress, anxiety, depression, and health distress were assessed using mixed effect models to account for repeated cortisol measures among participants [43]. Correlations between self-reported measures and HCC (only one measure per participant) were assessed using Pearson's correlation.

Intercept-only linear and log-binomial regression were used to estimate the overall and group-stratified mean and standard deviation or proportion for each 6-month follow-up cortisol measure, which accounted for the repeated cortisol measures from participants. For the group-stratified measures (i.e., intervention group and control group), separate models were run for each group.

Average differences in the cortisol biomarker measures (except for the ≥40% change in CAR) collected at 6-month follow-up between intervention and control group were assessed using linear regression; proportion of participants with a ≥40% change in CAR was assessed using log-binomial regression and reported as a prevalence ratio (PR). Because the intervention was randomized and no differences were seen in baseline characteristics across groups, all models were unadjusted, and only the 6-month values were used. For all salivary cortisol measures (i.e., except for HCC since only one measure was taken), repeated sampling was accounted for by using an autoregressive (AR1) correlation structure and robust sandwich estimators. An autoregressive correlation was chosen because it allows measures taken closer to each other (e.g., Day 1 and Day 2, versus Day 1 and Day 3) to be more correlated. As a sensitivity analysis, other correlation structures (e.g., compound symmetric) were used but had minimal impact on findings.

Based on the literature [36,44], prior to correlational analyses and modeling, CAR slope, change in CAR, and cortisol AUCg were log-transformed.

## 3 Results

A total of 103 participants were recruited to participate in the study. At baseline, 92/103 (89%) provided saliva for CAR and 54/103 (52%) provided hair for hair cortisol concentration. At 6-month follow-up, 78/103 (76%) provided saliva for CAR and 57/103 (55%) provided hair for hair cortisol concentration [25]. Of 103 participants, 27 were excluded (8 did not complete the follow-up survey and 19 did not provide samples with sufficient volume for assays or within the ± 15 collection time window for the 30-min post-awakening samples), leaving 76 women (34 from the intervention and 42 from the control group) that were included in these analyses. No statistically significant differences in patient demographics or breast cancer characteristics were observed between women who were included versus excluded from analysis (data not shown).

The median age of participants was 57 years (Table 1). Nearly all (93%) were Mexican immigrants and most (58%) identified Spanish only as their preferred language. Over half were married (62%). Most (76%) had a high school education or less and a majority (76%) were unemployed. Approximately 34% of participants reported experiencing financial hardship in the past year. Thirty-three percent reported having poor/fair physical health and 23% reported having poor/fair mental health. The median time since breast cancer diagnosis was 2.5 years, with the greatest proportion (76%) of participants being diagnosed with invasive breast cancer and slightly less than half (44%) of those diagnoses being Stage II. Most (61%) participants received both chemotherapy and radiation treatment. At baseline, 83% (63) had completed active treatment (not including endocrine therapy), 13% (10) were missing this information, and 4% (3) were receiving chemotherapy. In addition, 43 (57%) of women were receiving endocrine therapy at baseline. There were no statistically significant differences between women in the intervention and control group on any of these characteristics.

**Table 1:** Baseline characteristics of participants in the Cortisol Study, Nuevo Amanecer-II Randomized Controlled Trial, 2016–2018 (N = 76).

	Overall		Intervention		Control		p-value
**Total, N**	76		34		42		–
**Age, median (IQR)**	57	(50, 63)	58	(52, 64)	53.5	(49, 60)	0.12
**Years in US, median (IQR)**	28	(18, 38)	27	(22, 40)	28.5	(15, 35)	0.42
Missing	7		1		6		
**Birthplace, n (%)**							0.06
United States	5	(6.6)	0	(0.0)	5	(11.9)	
Mexico	71	(93.4)	34	(100.0)	37	(88.1)	
**Preferred spoken/written language, n (%)**							0.77
Spanish only	44	(57.9)	21	(61.8)	23	(54.8)	
Mostly Spanish	19	(25.0)	9	(26.5)	10	(23.8)	
Spanish and English equally	7	(9.2)	2	(5.9)	5	(11.9)	
Mostly English	6	(7.9)	2	(5.9)	4	(9.5)	
English only	0	(0.0)	0				
**Married or living with partner, n (%)**	47	(61.8)	22	(64.7)	25	(59.5)	0.81
**Education, n (%)**							0.04
Elementary or less	25	(32.9)	16	(47.1)	9	(21.4)	
More than elementary to high school graduate	33	(43.4)	10	(29.4)	23	(54.8)	
More than high school graduate	18	(23.7)	8	(23.5)	10	(23.8)	
**Employed, n (%)**	18	(23.7)	8	(23.5)	10	(23.8)	1.00
**Financial hardship within the past year, n (%)**	26	(34.2)	14	(41.2)	12	(28.6)	0.33
**Poor/fair self-rated physical health, n (%)**	25	(32.9)	14	(41.2)	11	(26.2)	0.22
**Poor/fair self-rated mental health, n (%)**	21	(28.0)	13	(39.4)	8	(19.0)	0.07
**Breast cancer diagnosis, n (%)**							0.99
Ductal carcinoma in situ	7	(9.9)	3	(10.0)	4	(9.8)	
Invasive	54	(76.1)	23	(76.7)	31	(75.6)	
Inflammatory	10	(14.1)	4	(13.3)	6	(14.6)	
Missing	5		4		1		
**Breast cancer stage at diagnosis, n (%)**							0.61
Stage 0	3	(4.4)	2	(6.7)	1	(2.6)	
Stage I	22	(32.4)	11	(36.7)	11	(28.9)	
Stage II	30	(44.1)	13	(43.3)	17	(44.7)	
Stage III	13	(19.1)	4	(13.3)	9	(23.7)	
Missing	8		4		4		
**Breast cancer treatment, n (%)**							0.81
Chemotherapy only	8	(10.5)	5	(14.7)	3	(7.1)	
Radiation only	17	(22.4)	7	(20.6)	10	(23.8)	
Both chemotherapy and radiation	46	(60.5)	20	(58.8)	26	(61.9)	
No treatment	5	(6.6)	2	(5.9)	3	(7.1)	
**Years since cancer diagnosis, median (IQR)**	2	(0, 4)	2	(0, 4)	2	(0, 4)	0.97
**Corticosteroid use,**^a^**n (%)**	16	(21.1)	7	(20.6)	9	(21.4)	0.99

Abbreviations: IQR, interquartile range.

a Reported using a glucocorticoid or steroid drug (pills, inhalers, nasal sprays) for asthma, arthritis, or allergies at baseline.

### 3.1 Six-month follow-up salivary and hair cortisol measures (aim 1)

Overall, 582 saliva samples were obtained at follow-up (262 from intervention and 320 from control group), with 42% of women providing all nine salivary samples (intervention group: 44%; control group: 41%). Hair samples (and HCC measures) were obtained from 49 women (22 from intervention group and 27 from control group). The overall mean awakening cortisol was 2.17 ng/mL (SD 0.07) (Table 2). The overall mean CAR slope was 0.10 (SD, 0.37), mean cortisol AUCg was 11.34 (in thousands, SD 8.68), and HCC was 1751.6 pg/mg (SD 1148.6). The proportion of samples with a ≥40% change in CAR was 42%.

**Table 2:** Descriptive statistics for untransformed cortisol measures at 6-month follow-up in the Cortisol Study, Nuevo Amanecer-II Randomized Controlled Trial, 2016–2018 (N = 76).

	Participants, N	Samples, N	Mean	(SD)	Range
Awakening cortisol, ng/mL	76	199	2.17	(0.07)	0.06–3.88
CAR slope^a^^,^^b^	68	176	0.10	(0.37)	−2.33, 1.15
Cortisol AUCg^a^^,^^b^, thousands	68	147	11.34	(8.68)	0.93, 36.66
Hair cortisol concentration^c^, pg/mg	49	49	1751.6	(1148.6)	340.9, 6084.3
	**Participants, N**	**Samples, N**	**N**	**(%)**	**95% CI**
≥40% change in CAR^a^^,^^b^	69	178	75	(42.2)	34.6, 49.9

Abbreviations: SD, standard deviation; CAR, cortisol awakening response; AUCg, area under the curve with respect to ground.

a Participants were asked to provide salivary samples for 3 days. CAR slope and ≥40% change in CAR measures utilized awakening and 30-min post awakening cortisol values; cortisol AUCg measure utilized awakening, 30-min post awakening, and bedtime cortisol values.

b Intercept-only linear and log-binomial regression was used to estimate the mean (standard deviation) and proportion of each cortisol measure, which allowed for the accounting of repeated measures.

c Only one hair sample was collected per participant (intervention group: 22; control group: 27).

### 3.2 Correlations of baseline cortisol measures and self-reported stress and psychological distress (aim 2)

CAR slope was positively and moderately correlated with ≥40% change in CAR and cortisol AUCg (Table 3) HCC was not correlated with any of the other salivary cortisol measures. None of the cortisol measures were correlated with any of the self-reported measures of stress, anxiety, depression, and health distress. All the self-report measures were positively and strongly correlated with each other.

**Table 3:** Correlations between baseline cortisol and self-report measures in the Cortisol Study, Nuevo Amanecer-II Randomized Controlled Trial, 2016–2018 (N = 76).

	Stress (PSS-10)	Anxiety (BSI)	Depression (PHQ-8)	Health Distress	CAR slope	Cortisol AUCg	HCC mg/mL	≥40% change in CAR
**Self-report measures**								
Stress (PSS-10)	1.00							
Anxiety (BSI)	0.69 <0.0001	1.00						
Depression (PHQ-8)	0.69 <0.0001	0.77 <0.0001	1.00					
Health Distress	0.72 <0.0001	0.64 <0.0001	0.71 <0.0001	1.00				
**Cortisol measures**^a^								
CAR slope^b^	−0.04 0.57	−0.07 0.33	−0.10 0.19	−0.04 0.62	1.00			
Cortisol AUCg^b^, thousands	−0.22 0.005	−0.23 0.003	−0.22 0.004	−0.21 0.006	0.27 0.0004	1.00		
HCC, mg/mL^b^^,^^c^	−0.08 0.34	−0.04 0.69	−0.04 0.65	−0.17 0.05	−0.12 0.22	0.14 0.17	1.00	
≥40% change in CAR	0.10 0.19	0.03 0.68	−0.02 0.82	0.13 0.08	0.48 <0.0001	0.03 0.68	−0.08 0.42	1.00

Abbreviations: AUCg, area under the curve with respect to ground; HCC, hair cortisol concentration.

a Participants were able to contribute up to 3 days’ worth of cortisol samples across 3 days; similar estimates were obtained when correlation was estimated using mixed effect models and accounting for the repeated measures [43].

b All cortisol measures were log-transformed before assessing correlation.

c Only 49 participants provided hair samples (intervention group: 22; control group: 27); due to small sample size repeated measures among the other cortisol measures could not be accounted for.

### 3.3 Differences in cortisol measures by experimental group (aim 3)

Overall, we saw no differences in cortisol measures at 6-month follow-up between the intervention and control groups (Table 4).

**Table 4:** Cortisol measures at baseline and 6-months, stratified by intervention group, and mean differences at 6 months between intervention groups, ancillary Cortisol Study of NA-II, 2016–18 (N = 76.

	Intervention Group				Control Group						
	Baseline		6-month		Baseline		6-month		Difference at 6 months^a^		
	Mean	(SE)	Mean	(SE)	Mean	(SE)	Mean	(SE)	Mean Difference	(95% CI)	p-value
CAR slope^b^^,^^c^	0.16	(0.04)	0.11	(0.04)	0.13	(0.04)	0.09	(0.04)	0.001	(-0.005, 0.008)	0.68
Cortisol AUCg, thousands^b^^,^^c^	11.9	(1.05)	10.89	(1.06)	13.2	(0.92)	11.7	(0.98)	−0.15	(-0.42, 0.13)	0.29
HCC, pg/mg^c^^,^^d^	713.9	(150.15)	1695.8	(254.1)	603.8	(115.8)	1797.5	(218.0)	−0.11	(-0.48, 0.25)	0.54
	**N**	**(%)**	**N**	**(%)**	**N**	**(%)**	**N**	**(%)**	**PR**	**(95% CI)**	**p-value**
≥40% change in CAR^b^^,^^c^	38	(47.3)	38	(46.1)	36	(37.9)	37	(39.0)	0.87	(0.42, 1.77)	0.69

Abbreviations: SE, standard error; CI, confidence interval; PR, prevalence ratio.

a Differences in 6-month cortisol levels between the intervention and control group were calculated using linear (CAR slope, cortisol AUCg, HCC) and log-binomial (≥40% change in CAR) regression; repeated measures were accounted for using an autoregressive correlation structure.

b Cortisol was log-transformed before all biomarker measures were calculated.

c Participants provided up to 3 saliva samples at each time point; biomarkers were modeled as repeated measures and within-subject correlation was accounted for using an autoregressive correlation structure and robust sandwich estimators.

d Only 49 participants provided hair samples (intervention group: 22; control group: 27).

## 4 Discussion

This study aimed to characterize the diurnal cortisol profiles of Latina breast cancer survivors living in rural areas, using salivary and hair cortisol measures, and the correlations of these stress biomarkers with self-report measures of stress and psychological distress. In exploratory analyses, we assessed the effects of the stress management program on cortisol measures of stress. We looked specifically at CAR slope, ≥40% change in CAR, total cortisol output (AUCg), and hair cortisol concentration to characterize cortisol profiles and HPA axis function. This is the first study, to our knowledge, to report on mean salivary cortisol levels and patterns among rural Latina breast cancer survivors.

Mean awakening value was 2.17 (SD 0.07) ng/mL in our sample, which was low compared to other studies [45,46]. For example, Cohen et al. showed mean awakening values of 20.30 nmol/L (7.36 ng/mL) in an adult population. Women in our study presented with flatter CAR slopes compared to a healthy adult population [33] and adults with childhood adversity, a group with altered HPA-axis functioning [47]. Cortisol profiles of women in our study suggest HPA axis dysfunction that can result from chronic stress.

Only 42% of samples had adequate percent increase (defined here as ≥40% from awakening to peak), further evidence that our participants experienced abnormal stress reactivity likely due to experiences of chronic stress. Mean cortisol AUCg levels for our participants were at least 10-fold higher than those reported among Latinos and Black participants in another study, and women with early-stage breast cancer [19,45,48]. AUCg levels in our sample indicate that women experienced sustained higher levels of cortisol throughout the day. Studies in ovarian cancer patients have found high cortisol AUCg and even higher levels among advanced-stage cancer patients [49]. Similarly, HCCs were also elevated in our sample, signifying high chronic stress. We found that participants presented with overall elevated hair cortisol concentrations [28,34] that were approximately 7 times higher than those found in a study of stressed individuals [50].

In our study, we did not observe substantial correlations between stress biomarkers and self-report measures. However, this is not surprising given that a meta-analysis showed mixed relationships between biomarkers and self-reported measures of stress among those experiencing trauma or chronic stress [51,52], and a recent cross-sectional study of cortisol among Latina breast cancer survivors also showed mixed relationships [53]. We did observe moderate, positive correlations among the salivary cortisol measures, but there was no correlation between salivary and hair cortisol measures. This is likely due to the difference in duration of the data collection period [54]. The salivary sample is considered a short-term measure (over 36-h) and the hair sample a longer-term measure (over 1.5 months for 1.5 cm of hair used).

There were no significant differences at follow-up in stress biomarkers among the intervention compared to the wait-list control group. Work by others [55] has shown that mindfulness interventions may reduce cancer-associated blunting of the CAR profiles during the first 60 min of chemotherapy infusion, that is, the acute treatment phase. This highlights the nuances of selecting the correct window of opportunity for measuring improvements in stress biomarkers and important distinctions between acute and chronic stressors. In our study, the final 6-month assessment was performed three months post-intervention (completion of the 10-week stress management intervention) and women were on average 2.5 years post-diagnosis, therefore we may have missed any improvements in cortisol profiles that may have occurred immediately post-intervention, or alternatively, that the intervention was insufficient to produce and sustain for three months any measurable improvements in cortisol in such a vulnerable population.

Our findings that participants presented with abnormal CAR profiles is of concern given the intersecting vulnerabilities of women in our sample. A conceptual basis for how stress can impact cancer tumor biology [10,12,15,18] has been posited. Further evidence is needed to identify specific mechanisms by cancer type. In addition, HPA dysregulation can potentially worsen breast cancer prognosis in other ways besides tumor biology among highly vulnerable groups such as Latina breast cancer survivors living in rural areas. For example, Septhon et al. showed that flattening of the diurnal cortisol slope was a long-term prognostic indicator in humans in general [18], possibly due to immune system suppression, chronic inflammation, and comorbidity due to allostatic overload (the cumulative biological burden of chronic stress and life events) [56]. Abercrombie et al. found that women with metastatic breast cancer had flatter diurnal cortisol rhythms [17]. HPA dysregulation becomes more pronounced with the progression of cancer [57].

Limitations to this study include the small sample size, which limited power for a statistical test of differences by treatment arms. Furthermore, at baseline, 43 (57% of women were receiving endocrine therapy, which can affect the neuroendocrine system. Also, our sample constitutes a very vulnerable group, Spanish-speaking Latina breast cancer survivors living in rural areas and mostly of Mexican backgrounds, and findings may not generalize to other populations. However, it is possible that other vulnerable population experiencing chronic stress may also present with abnormal cortisol profiles so characterizing these groups is critical. To minimize participant burden, this study did not collect or control for all potential covariates, which include menstrual cycle, mode of awakening (alarm vs. spontaneous), etc. Lastly, this study was inclusive of participants with non-metastatic breast cancer only and did not exclude participants based on time since their last breast cancer diagnosis.

## 5 Conclusions

Biological stress profiles are relevant for understanding potential mechanisms of survival disparities among diverse breast cancer survivors, especially among women experiencing life-threatening illnesses and financial hardship. Our study was able to characterize cortisol profiles among an underserved, understudied, and underrepresented population, rural Spanish-speaking Latina breast cancer survivors. We found abnormal CAR profiles, which could have implications for their breast cancer-related outcomes. Future research is needed to better understand trajectories of cortisol profiles before, during, and after a cancer diagnosis among vulnerable populations, and their associations with breast cancer survival. Additionally, culturally tailored stress management interventions are needed to address breast cancer health disparities in vulnerable groups experiencing high levels of chronic stress. Finally, addressing the upstream social factors that contribute to stress, such as poverty, access to care, and racism/discrimination will be needed to eliminate health disparities among breast cancer survivors.

## Credit author statement

**Cathy Samayoa:** Conceptualization, Methodology, Formal analysis, Investigation, Resources, Writing - Original Draft, Writing - Review & Editing. **Veronica Santana-Ufret:** Methodology, Writing - Original Draft, Writing - Review & Editing. **Jasmine Santoyo-Olsson:** Software, Formal analysis**,** Resources, Writing - Original Draft, Writing - Review & Editing. **Paula D Strassle:** Software, Formal analysis, Writing - Original Draft, Writing - Review & Editing. **Anita Stewart:** Conceptualization, Methodology, Writing - Original Draft, Writing - Review & Editing. **Jackie Bonilla:** Writing - Original Draft, Writing - Review & Editing. **Cristian Escalera:** Writing - Original Draft, Writing - Review & Editing. **Rebecca M Mendez:** Methodology, Resources, Writing - Review & Editing. **Leticia Márquez-Magaña:** Conceptualization, Methodology, Resources, Writing - Review & Editing. **Carmen Ortiz:** Resources, Writing - Review & Editing. **Rachel M Ceballos:** Methodology, Resources, Writing - Review & Editing. **Anna Maria Nápoles:** Conceptualization, Methodology, Resources, Writing - Original Draft, Writing - Review & Editing.

## Disclaimer

The contents and views in this manuscript are those of the authors and should not be construed to represent the views of the National Institutes of Health.

## Declaration of competing interest

The authors declare that they have no known competing financial interests or personal relationships that could have appeared to influence the work reported in this paper.
